# Supplementary material for: Phytohormone profiling in an evolutionary framework
Source: Nat Commun. 2024 May 8;15:3875. doi: 10.1038/s41467-024-47753-z (PMC11079000; doi:10.1038/s41467-024-47753-z)
Supplement: Supplementary file 1 — Supplementary Information [file 41467_2024_47753_MOESM1_ESM.pdf]

Phytohormone profiling in an evolutionary framework

Vojtěch Schmidt[1,2], Roman Skokan[1], Thomas Depaepe[3], Katarina Kurtović[2], Samuel Haluška[1], Stanislav Vosolsobě[2], Roberta Vaculíková[1], Anthony Pil[3], Petre Dobrev[1], Václav Motyka[1], Dominique Van Der Straeten[3], Jan Petrášek[1,2]

[1] Institute of Experimental Botany of the Czech Academy of Sciences, Rozvojová 263, 165 02 Prague 6, Czechia
[2] Department of Experimental Plant Biology, Charles University, Viničná 5, 128 44 Prague 2, Czechia
[3] Laboratory of Functional Plant Biology, Ghent University, K.L. Ledeganckstraat 35, 9000 Ghent, Belgium

Supplementary Information

Supplementary Figure 1. Endogenous phytohormone compounds detected in the biomass of green algae and land plants and ethylene emanation.
Supplementary Figure 2. Endogenous phytohormone compounds detected in biomass and culture media of selected green algae, stationary vs. proliferative cultures.
Supplementary Figure 3. Relative errors of data from LC/MS-analyzed samples.
Supplementary Figure 4. Levels of ethylene emanation by selected green algae and land plants.
Supplementary Figure 5. Contamination test of analyzed strains.
Supplementary Figure 6. Phytohormone detection in axenic and contaminated strains.

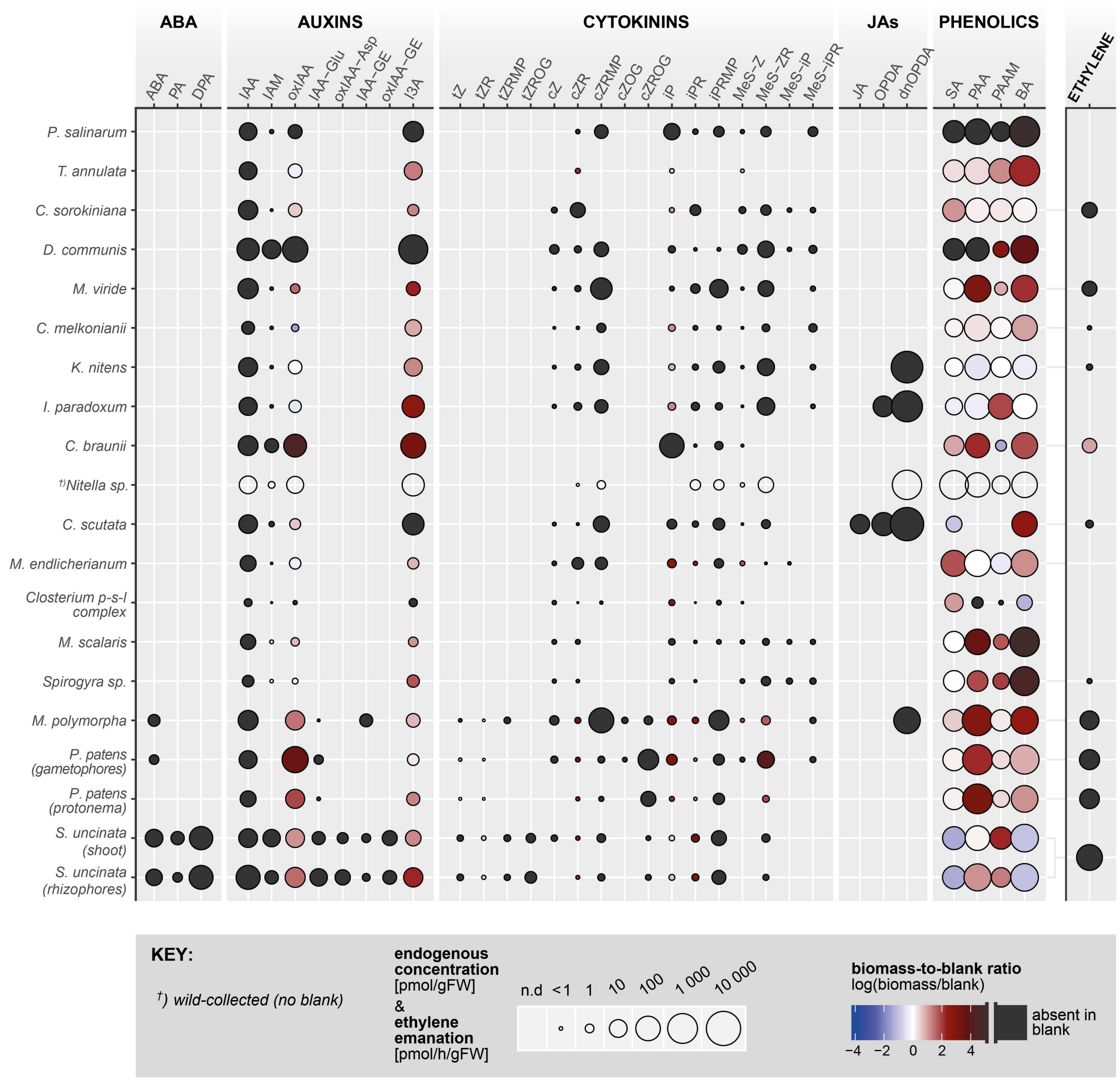

Supplementary Figure 1 | Endogenous phytohormone compounds detected in the biomass of green algae and land plants and ethylene emanation. Corresponds to Figure 1, but all analytes detected are listed. Circle size denotes mean concentration (pmol per gram fresh weight). No circle, compound not detected (n.d.). Color code denotes the ratio between the values measured in biomass and blank medium (containing no biological material), expressed in logarithmic scale: blue shading, compound(s) prevalent in blank; red shading, compound(s) prevalent in biomass; black, compound(s) absent in blank. Abbreviations: ABA (abscisic acid), PA (phaseic acid), DPA (dihydrophaseic acid), IAA (indole-3-acetic acid), IAM (indole-3-acetamide), oxIAA (2-oxo-IAA), IAA-Glu (IAA-glutamate), IAA-GE (IAA-glucose ester), tZ (*trans*-zeatin), cZ (*cis*-zeatin), Z(R)OGs (zeatin (riboside)-O-glucosides; both *cis*- and *trans*-isomers), iP (N^6-(Δ^2-isopentenyl)-adenine), MeS (methylthio), JA (jasmonic acid), OPDA (12-oxo-phytodienoic acid), dnOPDA (dinor-OPDA), SA (salicylic acid), PAA (phenylacetic acid), BA (benzoic acid). Minimum n=3 for biomass (independent cultures, each analyzed in technical duplicates; exact sample size for each strain is listed in Supplementary Data 3), n=2 for blank media. Ethylene measurements for both biomass and blank media were performed in n=5. Data variation is shown in Supplementary Figure 3 and Supplementary Figure 4 and listed in Supplementary Data 8.

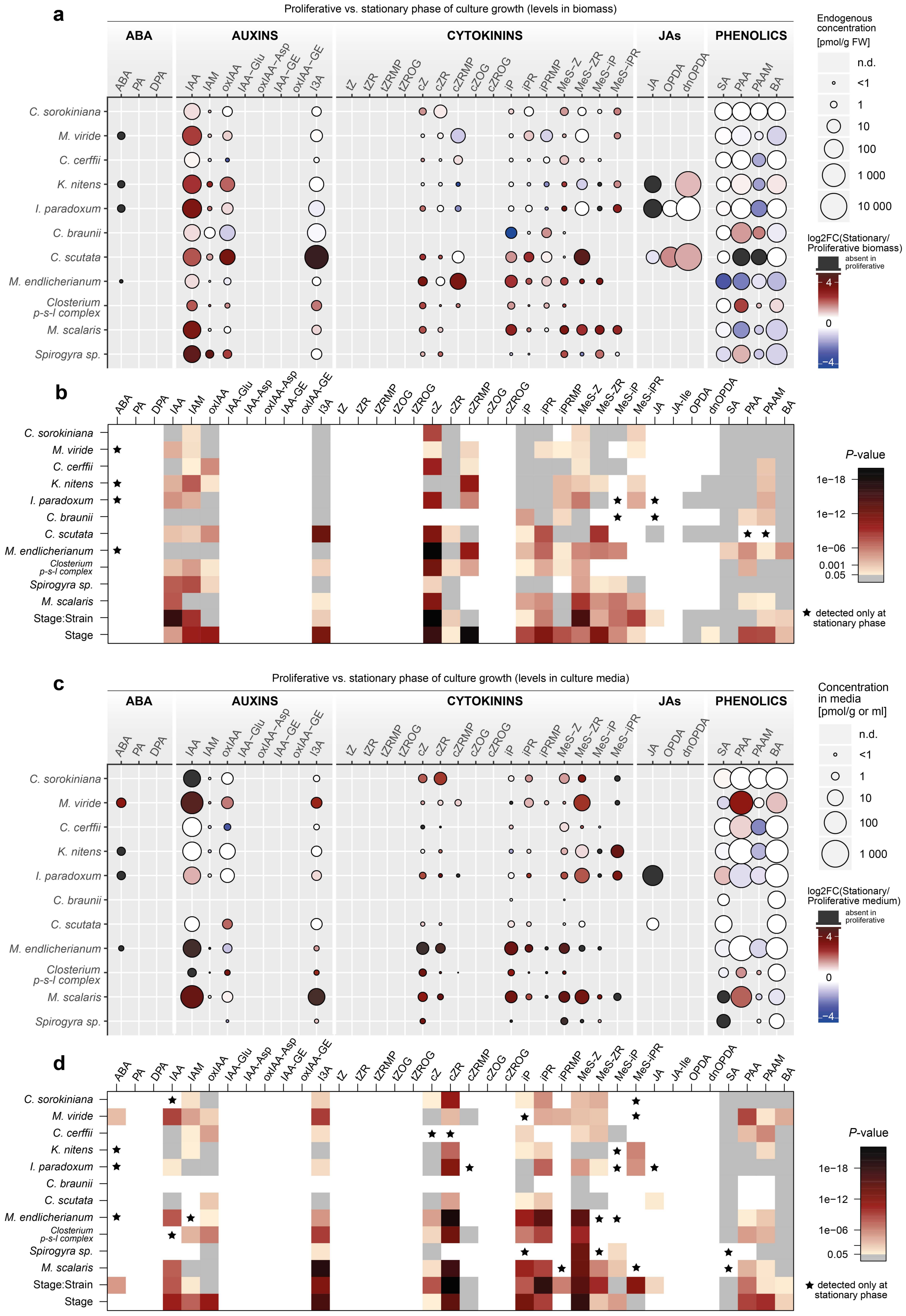
a
Proliferative vs. stationary phase of culture growth (levels in biomass)
ABA
AUXINS
CYTOKININS
JAs
PHENOLICS
ABA PA DPA
IAA IAM oxIAA IAA-Glu IAA-Asp oxIAA-Asp IAA-GE oxIAA-GE I3A
tZ tZR tZRMP tZROG cZ cZR cZRMP cZOG cZROG iP iPR iPRMP MeS-Z MeS-ZR MeS-iP MeS-iPR
JA OPDA dnOPDA
SA PAA PAAM BA
C. sorokiniana
M. viride
C. cerffii
K. nitens
I. paradoxum
C. braunii
C. scutata
M. endlicherianum
Closterium p-s-l complex
M. scalaris
Spirogyra sp.
Endogenous concentration [pmol/g FW]
n.d.
<1
1
10
100
1 000
10 000
log2FC(Stationary/ Proliferative biomass)
absent in proliferative
4
0
−4
b
ABA PA DPA IAA IAM oxIAA IAA-Glu IAA-Asp oxIAA-Asp IAA-GE oxIAA-GE I3A tZ tZR tZRMP tZOG tZROG cZ cZR cZRMP cZOG cZROG iP iPR iPRMP MeS-Z MeS-ZR MeS-iP MeS-iPR JA JA-Ile OPDA dnOPDA SA PAA PAAM BA
C. sorokiniana
M. viride
C. cerffii
K. nitens
I. paradoxum
C. braunii
C. scutata
M. endlicherianum
Closterium p-s-l complex
Spirogyra sp.
M. scalaris
Stage:Strain
Stage
P-value
1e−18
1e−12
1e−06
0.001
0.05
★ detected only at stationary phase
c
Proliferative vs. stationary phase of culture growth (levels in culture media)
ABA
AUXINS
CYTOKININS
JAs
PHENOLICS
Concentration in media [pmol/g or ml]
n.d.
<1
1
10
100
1 000
log2FC(Stationary/ Proliferative medium)
absent in proliferative
4
0
−4
d
P-value
1e−18
1e−12
1e−06
0.05
★ detected only at stationary phase

Supplementary Figure 2 | Endogenous phytohormone compounds detected in biomass and culture media of selected green algae, stationary vs. proliferative cultures. a Ratio between biomass in stationary and proliferative growth phases, logarithmic scale. Color code: blue shading, prevalent in proliferative phase; red shading, prevalent in stationary phase; black, absent in proliferative phase. Circle size denotes mean concentration in stationary cultures (pmol per gram fresh weight). Minimum n=3 for biomass and corresponding culture media (independent cultures, each analyzed in technical duplicates; exact sample size for each strain is listed in Supplementary Data 3). **b** Heatmap of significance levels for differences in endogenous concentration between proliferative and stationary phase. Log-transformed data were analyzed by linear mixed-effects model (two-sided hypothesis) and group differences were determined by multiple comparison (Tukey method, independently for each metabolite without additional adjustment). Overall significance of stage and its statistical interaction with strain identity are shown at the bottom of the heatmap. Color code: white, compound not detected; grey, $P > 0.05$; star, compound detected only at stationary phase. *P*-values are listed in Supplementary Data 9. **c, d** Same as (**a, b**, respectively), but in culture media. Circle size in (**a**) denotes concentration in stationary culture media (pmol per gram or ml). No circle in (**a**, **c**): compound not detected (n.d.). Compound abbreviations are listed in the legend to Supplementary Figure 1.

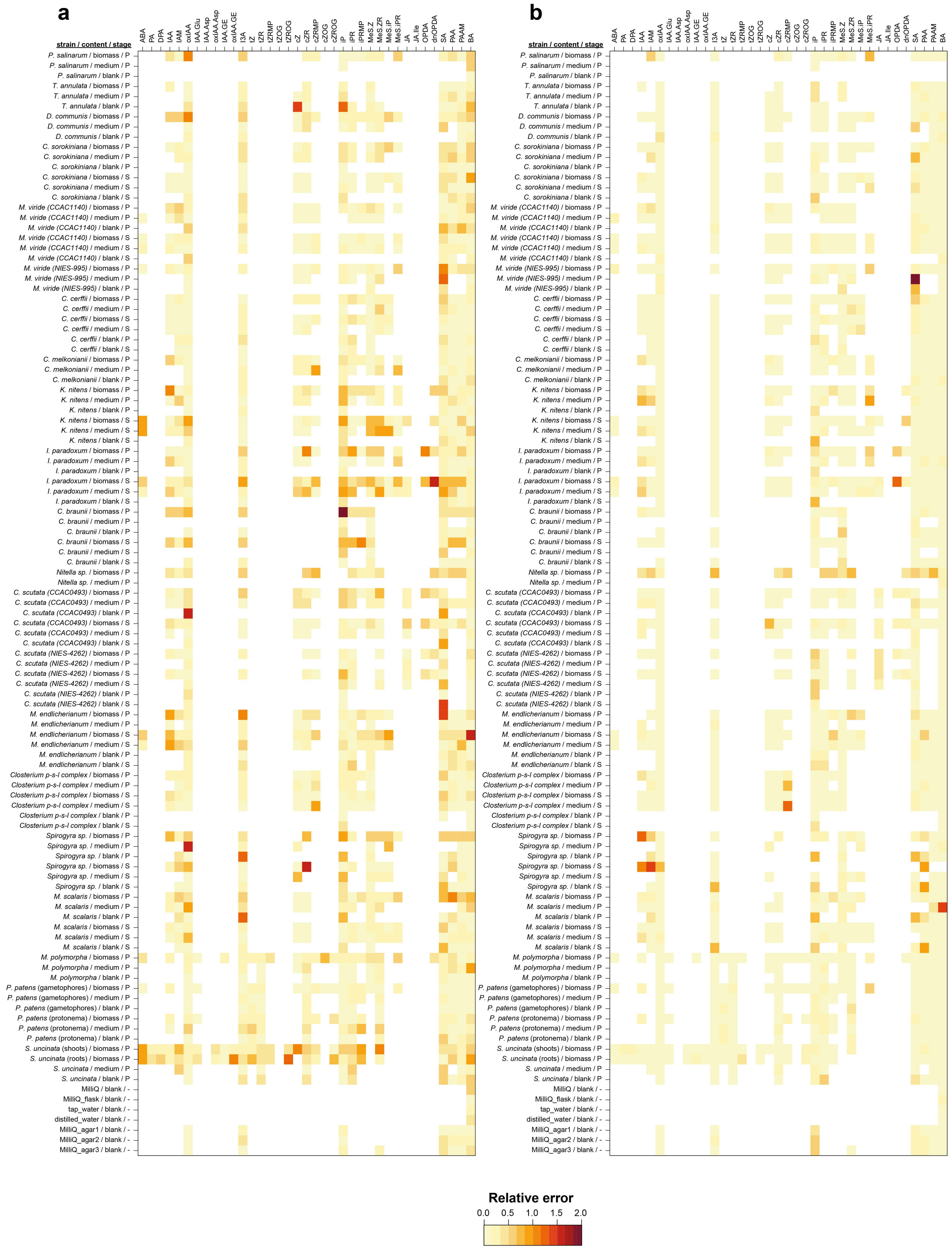

Supplementary Figure 3 | Relative errors of data from LC/MS-analyzed samples. a Heatmap of relative error of biological replicates; minimum *n*=3 for biomass and corresponding culture media (independent cultures; exact sample size for each strain is listed in Supplementary Data 3), *n*=2 for blank media. **b** Heatmap of relative error of technical duplicates. White fields indicate not detected analytes. Relative error values are listed in Supplementary Data 8.

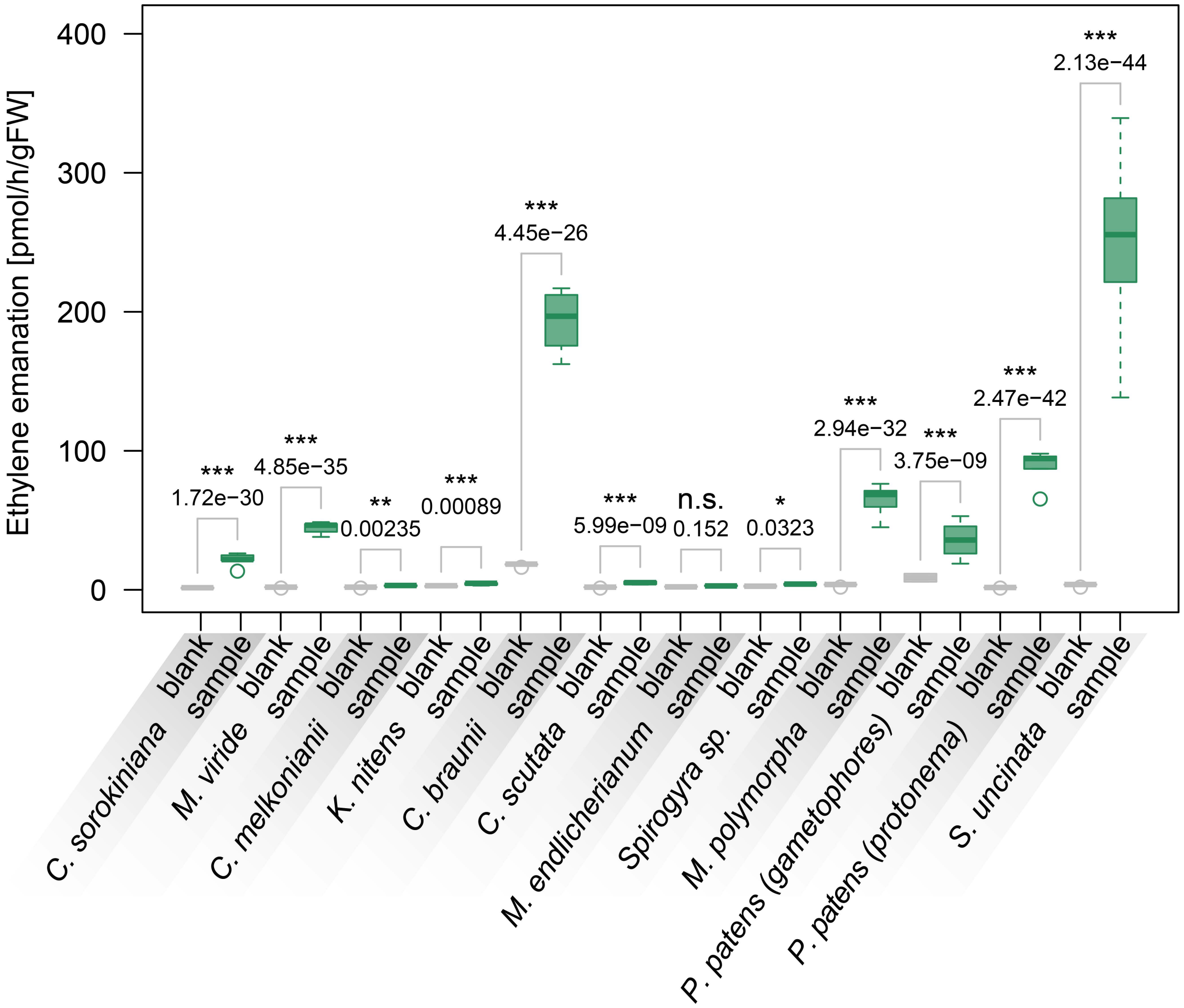

Supplementary Figure 4 | Levels of ethylene emanation by selected green algae and land plants. Data were obtained using laser-based photoacoustic spectroscopy. Green boxes represent living samples, grey boxes represent control media (blanks); boxes range between 1st and 3rd quartile, whiskers mark the 1.5 interquartile range. n=5 biological and blank media replicates (independent cultures). Log-transformed data were analyzed by linear mixed-effects model (two-sided hypothesis) and group differences were determined by multiple comparison (Tukey method, independently for each metabolite without additional adjustment), overall significance of blank-sample difference and its statistical interaction with strain identity was < 2e-16. Asterisks indicate significant differences between samples of tested living material and blank media background (*** $P < 0.001$, ** $P < 0.01$, * $P > 0.05$; n.s., not significant).

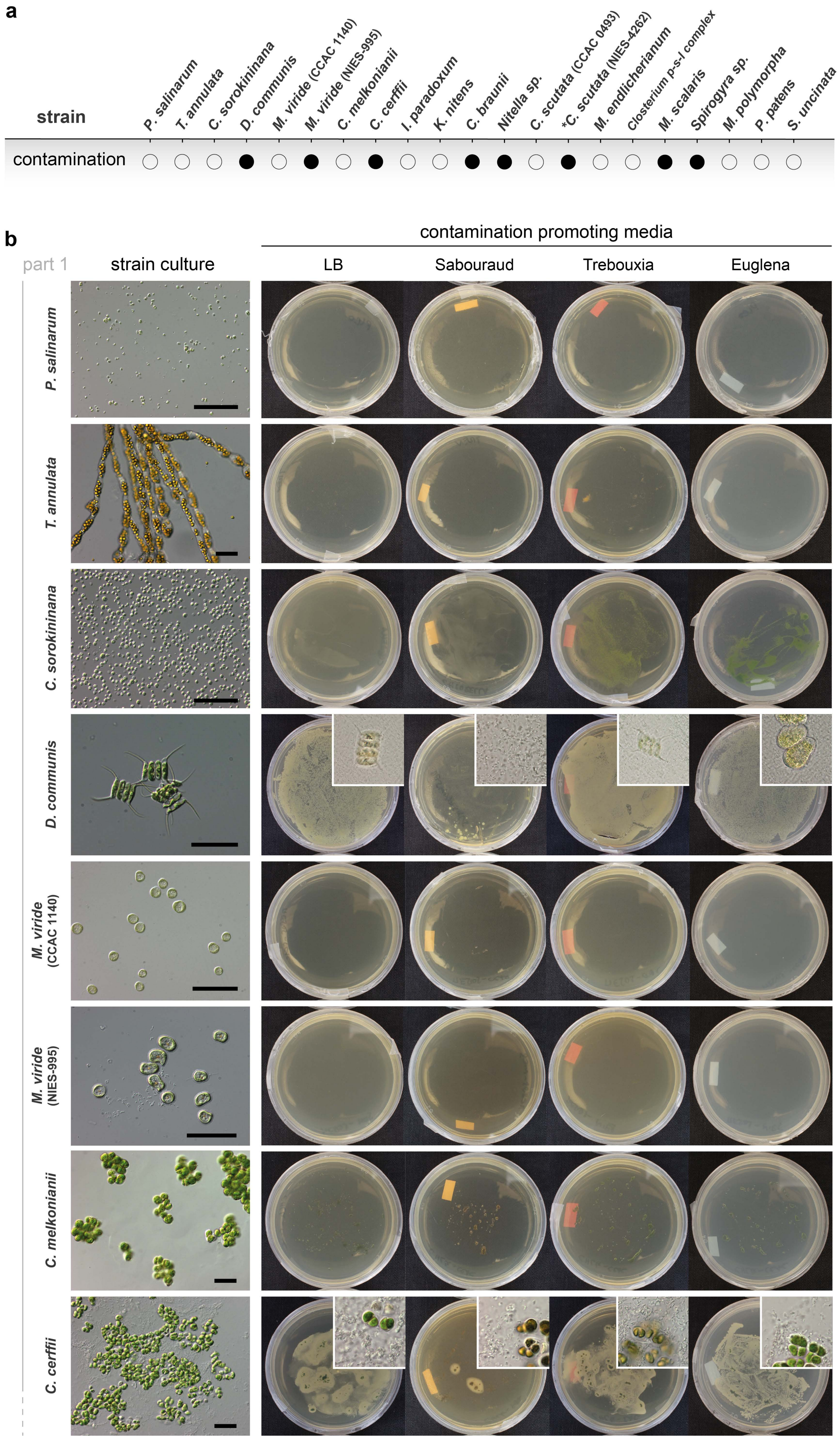
a
strain
P. salinarum
T. annulata
C. sorokininana
D. communis
M. viride (CCAC 1140)
M. viride (NIES-995)
C. melkonianii
C. cerffii
I. paradoxum
K. nitens
C. braunii
Nitella sp.
C. scutata (CCAC 0493)
*C. scutata (NIES-4262)
M. endlicherianum
Closterium p-s-I complex
M. scalaris
Spirogyra sp.
M. polymorpha
P. patens
S. uncinata
contamination
b
contamination promoting media
part 1
strain culture
LB
Sabouraud
Trebouxia
Euglena
P. salinarum
T. annulata
C. sorokininana
D. communis
M. viride (CCAC 1140)
M. viride (NIES-995)
C. melkonianii
C. cerffii

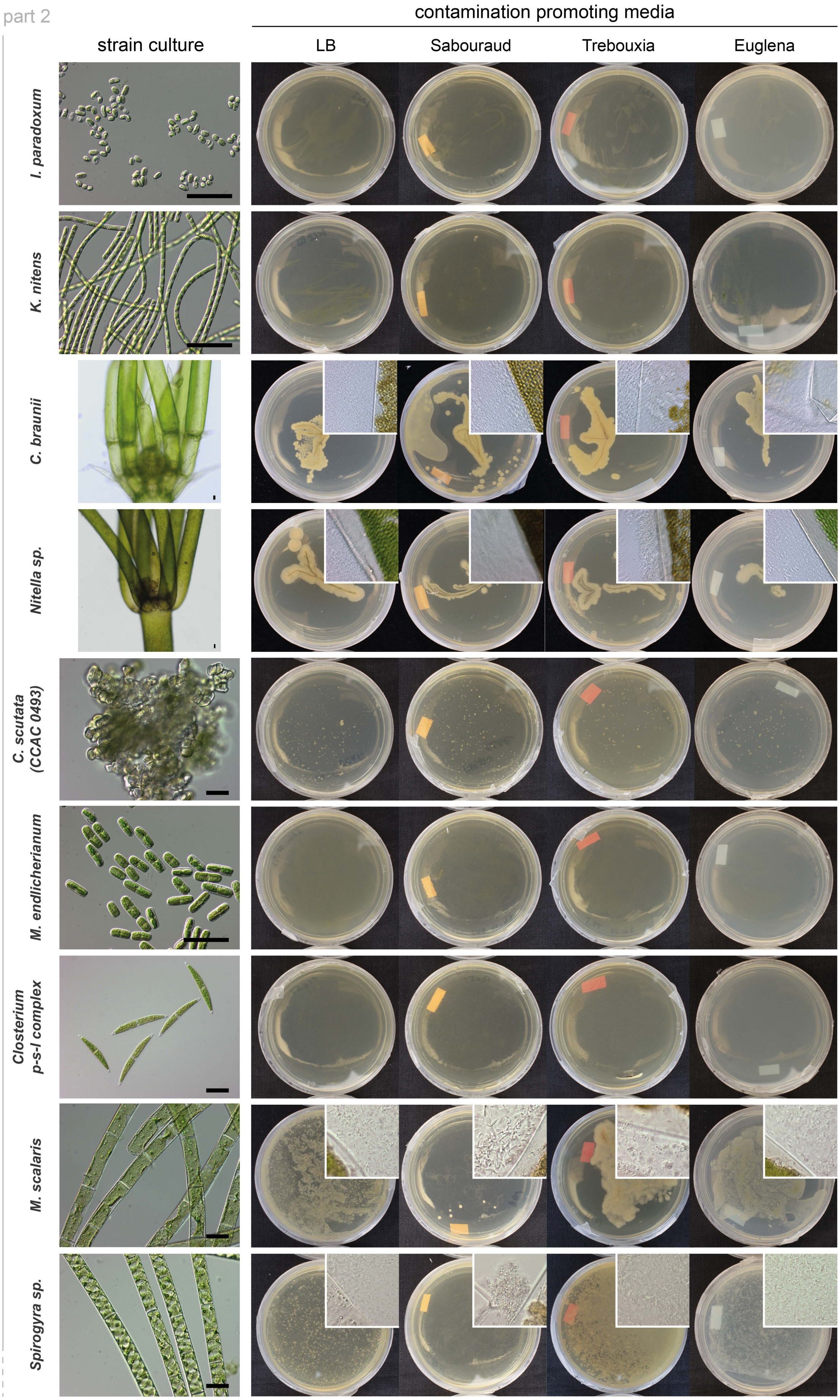
part 2
contamination promoting media
strain culture
LB
Sabouraud
Trebouxia
Euglena
I. paradoxum
K. nitens
C. braunii
Nitella sp.
C. scutata (CCAC 0493)
M. endlicherianum
Closterium p-s-l complex
M. scalaris
Spirogyra sp.

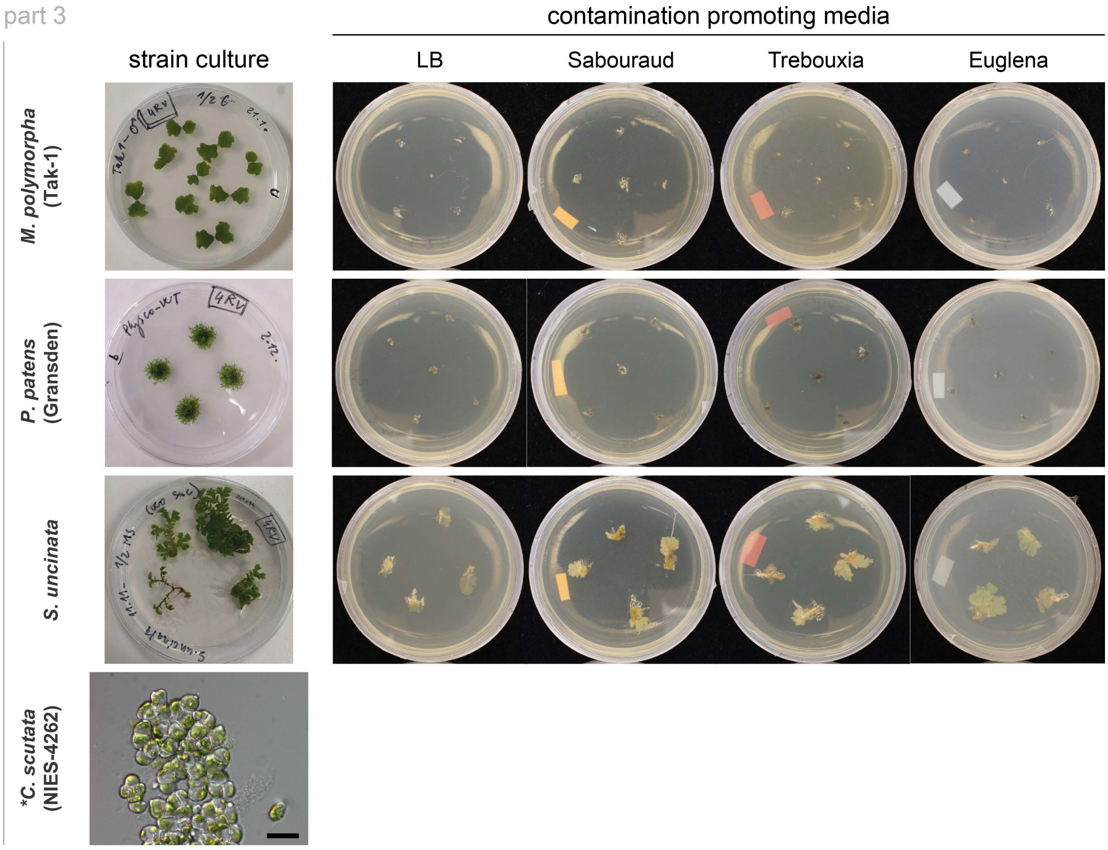

Supplementary Figure 5 | Contamination test of analyzed strains. a Summary of contamination in cultures of analyzed strains detected by microscopic examination and/or by sensitive test on contamination promoting media. Black circle, contamination detected; empty circle, contamination not detected. **b** Results of contamination test for individual strains. Left panel, images of strain cultures during sampling (Scale bar, 50 µm). Right panel, images of strains incubated in the dark at 37°C for 7 days on 4 types of contamination promoting media. Microscopic detail is provided for plates exhibiting contamination growth. Contamination-promoting media test was performed in 3 replicates with similar results. *) For *C. scutata* (NIES-4262), only microscopic examination was performed, as it was not available for the contamination-promoting media test.

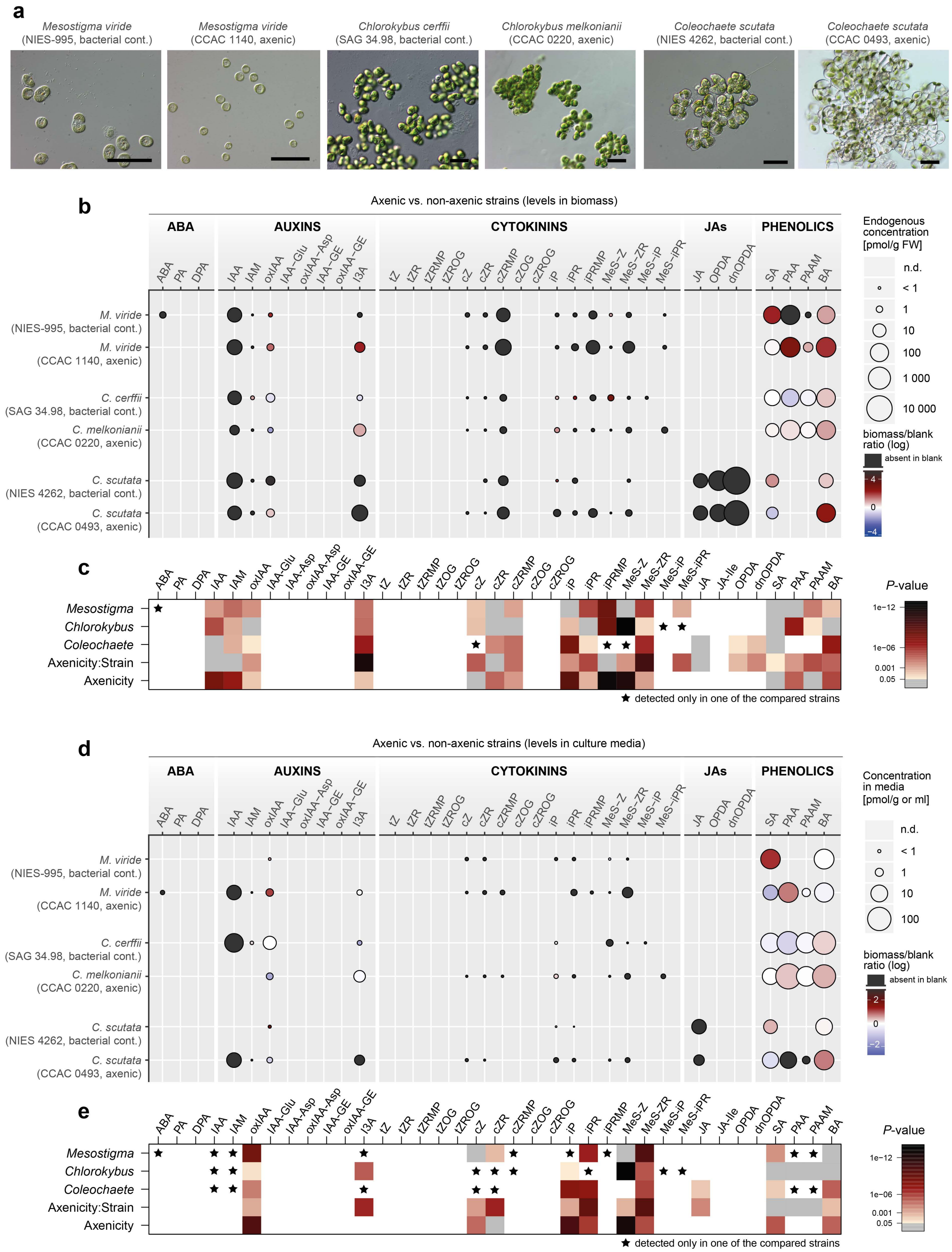

Supplementary Figure 6 | Comparison of phytohormone profiles in axenic and contaminated strains. a Microscopic images of axenic strains and strains with bacterial contamination (Scale bar, 50 µm). **b** Ratio between the values measured in biomass and blank medium (containing no biological material), expressed in logarithmic scale. Color code: blue shading, prevalent in blank; red shading, prevalent in biomass; black, absent in blank. Circle size denotes concentration in biomass (pmol per gram fresh weight). Minimum *n*=3 for biomass and corresponding culture media (independent cultures; exact sample size for each

strain is listed in Supplementary Data 3). **c** Heatmap of significance levels for differences in endogenous concentration between proliferative and stationary phase. Log-transformed data were analyzed by linear mixed-effects model (two-sided hypothesis) and group differences were determined by multiple comparison (Tukey method, independently for each metabolite without additional adjustment). Overall significance of axenicity and its statistical interaction with strain identity are shown at the bottom of the heatmap. Color code: white, compound not detected; grey, $P > 0.05$; star, compound detected only in one of the two compared strains. **d** Ratio between the values measured in culture media and corresponding blank media. Color code: blue shading, prevalent in blank; red shading, prevalent in biomass; black, absent in blank. Circle size denotes concentration in culture media (pmol per gram or ml). No circle in (**b,d**): compound not detected (n.d.). **e** Same as (**c**), but in culture media; *P*-values are listed in Supplementary Data 9. Compound abbreviations are listed in the legend to Supplementary Figure 1.
