# Supplementary material for: Phytohormone profiling in an evolutionary framework
Source: Nat Commun. 2024 May 8;15:3875. doi: 10.1038/s41467-024-47753-z (PMC11079000; doi:10.1038/s41467-024-47753-z)
Supplement: Supplementary file 3 — Description of Additional Supplementary Files [file 41467_2024_47753_MOESM3_ESM.pdf]

Description of Additional Supplementary Files:

Supplementary Data 1: List of analyzed strains.

Supplementary Data 2: List of analytes.

Supplementary Data 3: LC/MS raw data.

Supplementary Data 4: Ethylene spectroscopy raw data

Supplementary Data 5: Phytohormone levels in literature.

Supplementary Data 6: List of chemicals.

Supplementary Data 7: Illumination specification.

Supplementary Data 8: LC/MS data variation.

Supplementary Data 9: P-values for effects of culture conditions
