# Supplementary material for: Phytohormone profiling in an evolutionary framework
Source: Nat Commun. 2024 May 8;15:3875. doi: 10.1038/s41467-024-47753-z (PMC11079000; doi:10.1038/s41467-024-47753-z)
Supplement: Supplementary file 13 — Reporting Summary [file 41467_2024_47753_MOESM13_ESM.pdf]

nature portfolio

Corresponding author(s): Jan Petrášek, Roman Skokan

Last updated by author(s): Mar 21, 2024

Reporting Summary

Nature Portfolio wishes to improve the reproducibility of the work that we publish. This form provides structure for consistency and transparency in reporting. For further information on Nature Portfolio policies, see our Editorial Policies and the Editorial Policy Checklist.

Statistics

For all statistical analyses, confirm that the following items are present in the figure legend, table legend, main text, or Methods section.

n/a	Confirmed	
☐	☒	The exact sample size (*n*) for each experimental group/condition, given as a discrete number and unit of measurement
☐	☒	A statement on whether measurements were taken from distinct samples or whether the same sample was measured repeatedly
☐	☒	The statistical test(s) used AND whether they are one- or two-sided<br>*Only common tests should be described solely by name; describe more complex techniques in the Methods section.*
☒	☐	A description of all covariates tested
☐	☒	A description of any assumptions or corrections, such as tests of normality and adjustment for multiple comparisons
☐	☒	A full description of the statistical parameters including central tendency (e.g. means) or other basic estimates (e.g. regression coefficient) AND variation (e.g. standard deviation) or associated estimates of uncertainty (e.g. confidence intervals)
☐	☒	For null hypothesis testing, the test statistic (e.g. *F*, *t*, *r*) with confidence intervals, effect sizes, degrees of freedom and *P* value noted<br>*Give P values as exact values whenever suitable.*
☒	☐	For Bayesian analysis, information on the choice of priors and Markov chain Monte Carlo settings
☐	☒	For hierarchical and complex designs, identification of the appropriate level for tests and full reporting of outcomes
☒	☐	Estimates of effect sizes (e.g. Cohen's *d*, Pearson's *r*), indicating how they were calculated

Our web collection on statistics for biologists contains articles on many of the points above.

Software and code

Policy information about availability of computer code

Data collection	Agilent MassHunter B.08
Data analysis	Agilent MassHunter B.08, Microsoft Excel 365, R software package 4.2.3

For manuscripts utilizing custom algorithms or software that are central to the research but not yet described in published literature, software must be made available to editors and reviewers. We strongly encourage code deposition in a community repository (e.g. GitHub). See the Nature Portfolio guidelines for submitting code & software for further information.

Data

Policy information about availability of data

All manuscripts must include a data availability statement. This statement should provide the following information, where applicable:

- Accession codes, unique identifiers, or web links for publicly available datasets
- A description of any restrictions on data availability
- For clinical datasets or third party data, please ensure that the statement adheres to our policy

The source data for Figs. 1-2, and Supplementary Figs. 1-4, 6 are provided in the Source Data file. All raw LC/MS data generated in this study have been deposited in the Zenodo repository: 10.5281/zenodo.10411071.

Research involving human participants, their data, or biological material

Policy information about studies with human participants or human data. See also policy information about sex, gender (identity/presentation), and sexual orientation and race, ethnicity and racism.

Reporting on sex and gender	n/a
Reporting on race, ethnicity, or other socially relevant groupings	n/a
Population characteristics	n/a
Recruitment	n/a
Ethics oversight	n/a

Note that full information on the approval of the study protocol must also be provided in the manuscript.

Field-specific reporting

Please select the one below that is the best fit for your research. If you are not sure, read the appropriate sections before making your selection.

[x] Life sciences [ ] Behavioural & social sciences [ ] Ecological, evolutionary & environmental sciences

For a reference copy of the document with all sections, see nature.com/documents/nr-reporting-summary-flat.pdf

Life sciences study design

All studies must disclose on these points even when the disclosure is negative.

Sample size	Given the extensive scale of our analysis, a default minimum sample size of n = 3 (biological replicates) was established for biomass and culture media samples (independent cultures), while n = 2 was designated for control media (blank medium replicates) due to their absence of biological input. All LC/MS-analyzed samples underwent technical duplication. For ethylene measurements, all samples were analyzed in n = 5 (biological replicates) without technical replicates. Sample size was not pre-determined statistically.
Data exclusions	In adherence to data integrity, we have selectively excluded certain values from our study solely in instances where technical failures occurred (i.e. low abundance of internal standards during LC/MS analysis). Additionally, we have omitted values attributed to background noise (i.e. not detected analytes in the LC/MS analysis).
Replication	We can confirm that replication attempts were largely successful, except instances where technical failures occurred specifically during the LC/MS analysis, which lead to omission of corresponding values.
Randomization	Sampled cultures of each individual strain constituted its own experimental group. Sampling randomization was not relevant, as this study's objective was to profile numerous strains in standard conditions, rather than subjecting a single strain to varied conditions.
Blinding	Each sample was assigned a blinded numerical code prior to analysis to migitate any bias during analytical data quantification and evaluation.

Reporting for specific materials, systems and methods

We require information from authors about some types of materials, experimental systems and methods used in many studies. Here, indicate whether each material, system or method listed is relevant to your study. If you are not sure if a list item applies to your research, read the appropriate section before selecting a response.

Materials & experimental systems

n/a	Involved in the study	
[x]	[ ]	Antibodies
[x]	[ ]	Eukaryotic cell lines
[x]	[ ]	Palaeontology and archaeology
[x]	[ ]	Animals and other organisms
[x]	[ ]	Clinical data
[x]	[ ]	Dual use research of concern
[ ]	[x]	Plants

Methods

n/a	Involved in the study	
[x]	[ ]	ChIP-seq
[x]	[ ]	Flow cytometry
[x]	[ ]	MRI-based neuroimaging
